# On the Management of Fractures of the Extremities

**Published:** 1836-04-01

**Authors:** 


### ON THE MANAGEMENT OF FRACTURES OF THE EXTREMITIES.

I. Observations on Fractures. By *John Houston*, M.D. &c. &c. &c.*

II. On the Treatment of Fractures without Splints.

This is, in sober sadness, a most revolutionary age. Every thing is to be pulled down—nothing is to be built up. As Mr. Reeve says, when he deplores the diminution of executions at the Old Bailey, we are losing all our amusements. Surely it is bad enough to attack the institutions of the country—to undermine the colleges—to sap the hospitals—all this is bad enough, without attempting to destroy our fracture-boxes. But so it is. Surgeons have been groping for centuries in the dark in their management of fractures. They have supposed, silly people, that the ends of fractured bones are apt to ride, when in fact they themselves were riding their hobbies. All the pother about muscular contraction has been a brutum fulmen —a phantom of the imagination. Muscular contraction is the most obliging thing one can imagine—you ask it politely to be quiet, and it is so. Such are the new lights. They are brilliant in their way, but before we say any more about them, we will just hear what one of the old school has to say. Mr. Houston has not discovered that splints are useless, and accordingly we shall introduce him first, and listen to his funeral *éloge* of these departing instruments.

The object of Dr. Houston's paper may be briefly stated in his own words. "There are," he says, "in this country, two very opposite courses followed in the treatment of fractures of the lower extremities; one, which consists in laying the broken limb, during the first few days after the accident, in a flexed position, and, subsequently, either preserving the same posture all through the cure, or, after a given period, changing the bent condition of the limb for the straight posture: the other, that of placing the limb at once

---

* Dublin Journal, Jan. 1836.

in the extended position, and applying, from the onset, an apparatus destined to keep it, all through the period of healing, immoveably in that state." In order to point out the fractures of the leg adapted for the straight, and those calculated for the flexed position, Dr. Houston relates a series of cases, and appends to them the inferences he has been induced to draw from them. The cases detailed are fourteen in number. Of these eight are instances of fracture of the leg—three of fracture of the femur—two of fracture of the humerus just above the elbow-joint—and one of many fractures in succession. The majority of these cases present no features to arrest our attention. We shall merely notice the three following, each of which displayed some peculiarity.

Case 1. *Fracture of the Thigh—Non-union—Delirium Tremens.*

M'Auley, ætat. 45, addicted to frequent excesses in drinking, was admitted into hospital on the 22nd of April, 1834, with a fracture of the middle of the left thigh, the consequence of a fall. The limb was placed on a double-inclined plane of the most approved construction, and the utmost pains were taken to preserve it in a proper and steady position. There was considerable swelling and painful starting of the limb for several days. These symptoms, however, all subsided, but the patient remained restless and uneasy, and was much dissatisfied with his position. At the expiration of a month there was no sign of union, and even six weeks elapsed without any apparent advance being made towards a consolidation of the fragments, although the man did not exhibit any indications of derangement in his general health.

The limb was now placed in the extended position; strong adhesive plaster was applied round the broken part, and some blue-pill administered. A fortnight more elapsed, and still the broken pieces admitted of motion on each other. At this period a new and unexpected affection showed itself, namely, delirium tremens, in a paroxysm of which the man pulled off the splints, and leaped out of bed. In the effort at standing the limb yielded to the weight of the body, and was a second time completely bent at the fractured part. Having recovered from the delirium, the patient was removed to his own residence for change of air, and there, when he began to hobble about on stilts, having the thigh well braced with plaster and bandage, the union of the fracture was after a few months accomplished.

Dr. Houston offers the following remarks on the preceding case. We present them, because we intend to join issue with the Doctor.

" The failure in the process of union was not, here, for want of a proper apposition in the fragments of the bones, for the natural length and shape of the limb was not deranged; I conceive it to have been the result of the preference given in this case to the plan of treatment by the double-inclined plane, over that wherein the extended posture and straight splints are had recourse to. The grounds on which this opinion is founded are stated at a subsequent page.

The supervention of that affection, marked by all the symptoms which characterize the delirium of drunkards, and relieved by the treatment appropriate thereto, at so late a period as eight weeks from the time at which the man had totally given up the use of ardent liquors, and during all which he had never manifested any longing for indulgence in his favourite beverage, is, I believe, an unusual occurrence; and, that the affection under which this man laboured was none other than that just stated, may be gathered from the fact that he had previously had more than one attack of a similar nature, brought on by excesses in the use of ardent spirits. It is certain, however, that while in the hospital he had no opportunity of being supplied with such potations." 474.

We shall soon have occasion to find that Dr. Houston is a warm advocate for the plan of extension in fractures of the lower limbs. He is therefore not unlikely to exert some vigilance in the discovery of defects in the opposite method—we mean, in that of flexion. Such vigilance appears to us to be exhibited with more ingenuity than strict fairness in this instance. We cannot agree with him that the non-union may probably be laid to the account of the double-inclined plane. That method is constantly employed without being followed by any such results. In the apparatus of Mr. Amesbury, an apparatus of great use in cases of non-union, the principle of the double-inclined plane is adopted. We are ourselves strong advocates for extension, and for the use of the splint of Dessault, and therefore our defence of the double-inclined plane is perfectly disinterested. To "give a dog a bad name," is too often the practice, and although Dr. Houston is unconsciously unjust, still we do think that he is unjust towards the unfortunate apparatus we are speaking of.

We believe that the cause of non-union in this case was quite independent of local management. The patient was accustomed to large quantities of stimulus. Dr. Houston is at pains to inform us, that in the hospital that stimulus was withheld. The constitution was called on to do hard work in the reparation of the fracture—and it failed. This is no hypothetical view, but founded on facts which have come under our observation.

A man was under our care, as house-surgeon of St. George's hospital, on account of simple fracture of both bones of one leg. We laid the limb upon the side, on a splint, and made pressure by means of another side-splint and bandages. At the end of six weeks, we found, to our surprise, that the union was so soft, as to allow of flexion of the bones at the seat of fracture. We now inquired with more minuteness into the habits of the patient, and we found that he had been accustomed to drink several pots of porter daily. We had previously allowed him the ordinary diet of the hospital, and a pint of porter at his dinner. We now gave him three pints during the day, and put up the limb again as before. Ossific union rapidly ensued, and the patient was discharged in two or three weeks more.

Another man was under our care with compound fracture of both bones of one leg. Some time had elapsed, but there was no appearance of union. We learnt that the man, who was a "waterman"* at a hackney-coach-stand, was in the habit of drinking enormous quantities of gin. His favourite beverage was liberally prescribed, and the improvement was instantaneous.

A woman was received into the hospital with simple fracture of one leg. Violent spasms of the limb ensued. To relieve these, the house-surgeon, under whose charge she was placed, bled her freely. She had previously been accustomed to a full diet and to porter, and the consequence of the depletion was much debility. The fracture did not unite, and although various means, among others the seton, and cutting down upon the fractured

---

* These people, we presume, are called "watermen" ut lucus à non lucendo, because they drink no water.

ends of bone, have been employed, the fracture continues ununited by osseous matter.

We think that these cases, and other illustrations of the fact might be adduced, will render it probable that the want of union, in Dr. Houston's patient, was principally owing to the abstraction of his accustomed stimulus. That view of the case is strengthened by the occurrence of delirium tremens. We are much astonished at the remarks of Dr. Houston on this subject. He appears to dwell on the singularity of the supervention of delirium tremens, at a time when the patient was *not* indulging in the use of ardent spirits. This is commonly the case. The immediate cause of delirium tremens is most generally the sudden deprivation of the stimulus to which the individual had been previously habituated. Now and then, delirium tremens occurs while the person is committing excesses in drinking, but most usually, we repeat, it is after a cessation, a sudden cessation, of habitual indulgence, and not actually during the indulgence, that this peculiar affection is observed. This is so notoriously true of the form of delirium tremens that succeeds to injuries, that, in the London hospitals, we always inquire into the habits of the patients, and make a point of giving them, at the very earliest opportunity, the stimulating beverage to which they are accustomed. We should say that this is one of the great practical improvements of the day, and we would recommend Dr. Houston to look more carefully into the subject than he would appear to have hitherto done.

The next case is of a very different character—it is an instance of a great liability to fracture on the part of a child.

CASE 2. Patrick O'Hara, ætat. 5, an extremely handsome, chubby boy, and born of healthy parents of large stature, was brought into hospital on the 14th of January, 1834, with fracture of the left tibia, produced by a fall, in running to escape from a dog which frightened him. His mother stated that the opposite leg had been broken in the same place, and from an equally trifling cause, about nine months before, and examination proved the truth of her statement, for, not only had it been broken, but bore evidence of a bad job in the reparation. The recent fracture became united, and the patient was dismissed from hospital by the end of the third week.

On the 26th of November, in the same year, he was brought back by his mother, in equally apparent good health, with a fracture of the middle of the right thigh bone, caused by a fall of the same simple description as in the former instance. From this accident he recovered as expeditiously and as completely as from that of the tibia, and went home well. On the 13th of August in the following year, he was again taken to the hospital, with fracture of the right femur. From this he recovered well, and is at present healthy and strong. The bones are all straight—the joints well knit—and there is no apparent disease in the osseous system.' It seems clear that the bones are more than usually brittle, and that is all that can be said.

The third and last case to which we shall direct attention, is one of fracture of the femur, immediately beneath the lesser trochanter.

CASE 3. Ellen Kenny, æt. 13, was admitted June 17th, 1835, with fracture of the right thigh, just below the trochanter minor. It had happened two days before her admission, and the limb had been suffered to continue flexed in the interim. The superior portion of the bone was drawn upwards—the limb was greatly swollen and vesicated—and severe spasms were experienced. It was not deemed prudent to do any thing more than apply refrigerating lotions for the next three days, and, during this time, the spasms continued. On the sixth day after the occurrence of the accident, extension of the limb was made, and the limb then maintained extended by means of Dessault's splint on the outside, with a corresponding long splint on the inside, and a short splint in front, to compress the projecting upper portion of the femur. The operation of extension was painful, but the subsequent relief was great. The case did extremely well; and, on the 20th of August, the patient walked out of the hospital, without any appreciable difference in length, &c. between the two lower limbs.

We have introduced this case for the purpose of remarking that, although Dessault's splint answers admirably in cases of fracture of the thigh below its upper third, yet it does not generally succeed so perfectly, when the fracture is immediately below the trochanter. The reason is obvious to all who are familiar with the application of the splint. The counter-extension of the limb is effected by means of a bandage, passing from the notched summit of the splint, on the outside of the hip, to the commissure of the thigh and perineum. The effect of this is necessarily to pull the upper portion of the fractured thigh outwards, and this cannot well be prevented by any splint on the inside of the limb, because the latter splint cannot go higher than the perineum. If the fracture of the thigh is much below the level of the summit of the inner splint, the counter-extending bandage cannot force the fractured portions outwards, because the lateral compression of the two splints is sufficient to prevent it. In the case before us, the splint of Dessault *did* answer, notwithstanding the fracture was situated higher in the limb.

Let us now hear what Dr. Houston has to say on the management of fractures. The first point to which he adverts, is the occurrence of spasmodic contraction of the muscles.

"The time of accession of this distressing symptom is liable to some variety, but generally it appears in a few hours. It is most severely felt when the patient is falling asleep, causing him to start in a fright, deranging the broken fragments, and producing great pain. The least movement of the bed, or even the step of a by-stander on the floor, will often be productive of the same effect. The period of the duration of the spasms is not in all cases the same; they usually cease about the sixth or seventh day, but sometimes go on for a greater length of time. A case has been communicated to me by Mr. Cusack, in which they were extremely severe, and terminated on the sixth day, in the production of general tetanus, of which the patient lost his life. Spasms are usually most severe when the inflammatory action runs high; and this latter is no doubt aggravated by their presence.

The occurrence of spasms in a fractured limb would appear to be independent of any injury which the soft parts may have sustained from the accident. The largest flesh-wounds are exempt from spasms; whilst the simplest forms of complete fractures, those freest from lesion of the surrounding soft textures, are as liable as any others to be attended by this symptom. A spasmodic condition of all the muscles of a limb sometimes, it is true, follows a trifling wound of the flesh, uncomplicated with fracture of the bone, but this is a peculiar disease, not admitting of a comparison with that irregular spasmodic action usually attendant on fractures of the long bones of the extremities, nor relievable by the same means." 478.

Dr. Houston proceeds to discuss, at some length, the cause of spasms after fractures. Allowing something for the irritation of spicula, he argues that the principal agent in their production is the shortening of the limb, and the consequent loss of the fixed points of attachment of the voluntary muscles. He illustrates this opinion in a variety of ways. Perhaps one of the most striking analogical instances is that of the stump after amputation. The spasms that are well known to occur under these circumstances cannot be occasioned by the irritation of spicula of bone. This is true, no doubt; yet those muscles have been cut, and, if not irritated by bone, have been irritated by the knife. Probably Dr. Houston is correct, in attributing the production of the spasms principally to the shortening of the limb. Yet there have been a sufficient number of dissections to warrant us in believing that spicula of bone do occasionally give rise to spasms, and those of a very severe description. Did we trust our own observation, we should say that spasms, after fractures, are less common than the cases and remarks of Dr. Houston might lead an inexperienced surgeon to believe. At St. George's Hospital, we have seen only two or three cases of this kind within the last twelve years. At that hospital, fractures are usually "put up" at once, and the treatment by extension is most frequently, though by no means universally, adopted. If there is any irritability about the patient or the limb, a full dose of opium is generally given at once.

An acquaintance with the views of Dr. Houston, on the cause of spasmodic contractions of the muscles of the broken limb, would lead us to anticipate the following mode of treatment.

"With these facts so fully established, namely, that no degree of injury, unless accompanied by a fracture of a bone, is followed by spasms of the muscles, of the kind here alluded to—that no fractures of the bones in the body, except those of the extremities, give rise to those spasms—and that, even in the case of the extremities, if the broken fragments be naturally so circumstanced as not to admit of derangement from the action of muscles, there will be a complete exemption from unnatural spasmodic action, an obvious practical conclusion suggests itself, namely, that by imitating artificially the condition adverse to the development of spasms in either of these cases; viz. by restoring the broken fragments to their original places, and retaining them immoveably in that predicament, avoiding at the same time all hurtful pressure, a similar exemption from this harassing symptom may be obtained. Such anticipations are very plausible in theory, and will, I believe, if the experiment is properly made, be generally realized in practice." 483.

*Pain* is very commonly associated with spasms, and the same cause that produces one may excite the other. It appears to us, however, that the mere shortening of the limb is not, per se, likely to occasion pain. That is, in general, dependent on the position of the fragments, the laceration of the parts, the extension of the nerves and skin, &c. There can be no question that the proper reduction and coaptation of the fracture are the principal means for relieving the suffering, dependent essentially on displacement of the bone.

Dr. Houston's remarks on the local inflammation and general fever that succeed to fractures, offer nothing to detain us. It is obvious that the circumstances of the individual case must be investigated, in order to enable the surgeon to determine in each instance the causes in operation, and the remedies adapted for them.

Dr. Houston proceeds to consider the best mode of treating fractures, and the comparative advantages of the bent and straight positions. He repeats much of what we have already dwelt on, with respect to the dependence of spasm on shortening of the limb, or on relaxation of particular muscles. We must say that our author rides his hobby at a good round pace. Take, for example, the following case, adduced in confirmation of his views.

*Case.* " A young woman, labouring under white swelling, with ulceration of the cartilages of the knee, but in whom the joint was to a certain extent very moveable, on account of a relaxed state of the ligaments, and effusion into the cavity, became affected with the most excruciating spasms of the limb, accompanied with pain, which forced from her heart-rending screams. The flexor muscles appeared to be those most engaged in the spasms, as the knee, on every occasion of starting, was raised in a bent direction from the bed. She suffered in this way for nearly three weeks, during which sleep scarcely ever closed her eyes, even under the fullest doses of opium; for notwithstanding the soporiferous influence of this medicine, she was continually roused at the instant of slumbering by a violent spasm of the limb. Neither had local bleeding, counter-irritation, or the application of anodyne liniments, the least effect in appeasing her sufferings. Under these circumstances it was determined to try the experiment of treating the case as one of fracture attended with spasms. A tailed bandage was accordingly applied around the knee with uniform and moderate pressure, and long bran-pads and splints were so adjusted as to prevent the possibility of any motion in the joint or limb; abstaining at the same time from all other medicinal treatment. The practice was most successful; for, from the instant of its adoption, the spasms completely subsided, and all pain ceased. Under a continuance of this plan the patient lay contented and at ease, until anchylosis of the joint was accomplished." 486.

Now we greatly doubt whether this can be regarded as conclusive evidence on the side of the opinion, that it is extension of a limb which especially prevents spasm. When ulceration of the cartilage of an articulation has occurred, the slightest *motion* occasions pain, and commonly gives rise to spasmodic contractions of the muscles. So marked a symptom of ulceration of the cartilage is this, that it is insisted on as almost pathognomonic by Sir Benjamin Brodie and others. The way to prevent this is to prevent motion, and to maintain the joint in a state of perfect rest. In different joints the position best adapted for this purpose varies. The knee should be slightly, and very slightly, flexed—the elbow should be kept bent at a right angle. If Dr. Houston's theory were true, the position in the latter instance would be ill-calculated to obviate spasm. But it does obviate it notwithstanding; and the effects of a pasteboard splint which keeps the elbow bent, are as satisfactory as those of a corresponding splint, which keeps the knee-joint straight. It is the absolute rest obtained in each case, rather than the mere position, which, so far as we have seen, has proved of benefit.

Dr. Houston remarks, in continuation, that in the management of fractures, the bent position encourages the action of the flexors, already too disposed to contract inordinately. For this, and for the other reasons on which we have already dwelt, Dr. Houston announces himself the advocate of the extended posture in the treatment of fractures, accompanied, if necessary, with extension.

Dr. Houston recommends immediate "setting" of the limb, provided no particular circumstances are present to render it improper or unadvisable. We cordially agree with him. The limb is usually set with less trouble, less pain, and less extension in the first instance than afterwards, and the thing is done at once instead of at twice or thrice, or oftener. Dr. Houston urges many objections against the double-inclined plane in the management of fractures of the leg and thigh. We think with Dr. H. that it is a clumsy instrument—unnecessary, even if effective, in the former case—and possessed of many vices in the latter. Dr. Houston presents a detailed account of the apparatus employed by himself. It is sufficient for us to observe, that it consists of a long straight splint, resembling the splint of Dessault, applied on the outside of the limb—and of another long straight splint on its inside. These splints are properly padded, and to holes in each, at their lower extremities, a foot-board is adjusted. A tailed bandage is applied previously to the splints themselves. If extension is required, it is made by a bandage attached to the ankle, and the notch at the lower end of the outer-splint; while counter-extension is obtained by a bandage passing between the perineum and the notch at the upper end of the same splint.

Such are the essential features of the observations of Dr. Houston, as well as of his apparatus. The gist of the former is the recommendation of extension—while the latter is a modification of the apparatus of Dessault, employed at many of our hospitals, and more particularly at St. George's.

In principle we agree with Dr. Houston, but we think that his views are very incomplete in their details. We are quite satisfied, for our own parts, of the superiority of the plan of extending fractured limbs over that of humouring and bending them. Of this we are quite satisfied. But we are not so contented with Dr. Houston's one Procrustian method.

In the first place, the splint of Dessault, or modifications of it, do not answer well in *some* cases of fracture at or near the trochanters. The bandage of counter-extension occasionally interferes with the coaptation of the bone. In the majority of instances this objection can be obviated by making the long splint reach as high as possible, (almost to the axilla,) when the counter-extension is made more directly in the axis of the limb. Yet we have seen the long splint fail, and the double-inclined plane adopted of necessity, in some cases of fracture in this situation. On the whole, the Dessault's splint is an excellent apparatus for fractures of the thigh. A surgeon conversant with the mode of its employment, can prevent shortening and deformity of the limb with almost absolute certainty. The long splint on the inside of the limb is a very good addition, but it is not indispensable. A very good plan is to apply, besides the Dessault's splint on the outside of the thigh, three short splints—one on the inside of the thigh, one in the front, and one behind it.

But in fractures of the leg we are not disposed to approve equally of Dr. Houston's plan of management. There appear to us to be two objections against the long splint in fractures of the leg. In the first place it is unnecessary. There is no good reason that we can see for crippling the whole limb. Apply the long splint as well as it possibly can be applied, and still it is far from an agreeable apparatus. There must be some pressure on the perineum—the patient is absolutely stretched upon his back—and the passage of the stools and urine is troublesome. There are so many inconveniences of this description attendant on the long splint, that no patient would or should submit to them without the prospect of some great advantages. The inconveniences are not very great, but still they are inconveniences, and every unnecessary one, small as it may be, is no doubt discreditable to the surgeon. We have no hesitation whatever in asserting that fractures of the leg, simple or compound, may be well managed without the long splint, better indeed, in our opinion, than with it.

The second objection that we have towards the long splint, or any lateral splints whatever, whilst the limb is laid on its posterior surface, is its inapplicability to some cases, and the inadequate support to the back of the limb afforded in many others. We say its inapplicability to some cases. Suppose, what is not uncommon, a compound fracture with a low lateral wound. That wound cannot be dressed without daily disturbance of one splint, perhaps of both. That is a serious evil. There is nothing more clumsy, nor more mischievous, than repeatedly undoing an apparatus and trusting to the support afforded by the awkward or shaking hands of assistants. Even if the wound is not low on the side of the limb, but situated on the front of it, still, if there is much discharge, that cannot be prevented from running down between the splints and limb, rotting the paddings, and lodging behind. What are we to do then? There is a great complicated apparatus to change. A long splint is to be wholly or partially removed from the outside, and another must be quite taken away from the inside, and the cloth that connects them behind must be changed too. We are quite sure this is *not* a convenient method of treating compound fractures of the leg.

Then, again, in many cases, it affords an inadequate support to the broken limb. That support behind consists solely in the towel or band which passes from one splint to the other. There is little to prevent that from yielding to every possible degree, except the lateral compression of the side splints, and their fixture at the thigh and ankle. This cannot be so steady or secure as in many cases of fracture of the leg, and especially of compound fracture, is absolutely indispensable. We should say that, in compound fractures of the leg, the following are essential requisites for a really good apparatus—means of fixing perfectly the knee and foot, between which, when fixed, the extension is first made and afterwards maintained—good support to the limb in the intermediate points, that support being chiefly applied to the part of the limb, the back or the side, as circumstances may require, which we choose to be dependent—and, finally, a facility of getting at the limb, and thoroughly changing the dressings, without disturbing the fractured bones. For the reasons we have mentioned, and for others which we might advance, we do not conceive that the long splints proposed by Dr. Houston are calculated to answer the objects we have stated.

In simple fractures of the leg, attention and skill will make almost any apparatus answer. At all events, they usually will do so. We have seen the common side splint—the junk—Mr. Amesbury's ingenious apparatus—the swing German apparatus—the fracture-box of Assalini, &c. succeed almost equally well. The great point is to procure good apposition of the fractured surfaces at first, and to watch the limb with gentle assiduity. We do not therefore attach a very high degree of consequence to the *kind* of apparatus in the management of simple fractures. Much more depends on the knowledge of the surgeon. We must say that the position of the limb on the outside, laid in a well-shaped wooden splint with a foot-piece, and secured to it in such a way at the knee and ankle, that the many-tailed bandage can be used between those points—we must say that this outside splint conjoined with a common splint on the inside, so applied as to be readily removed without disturbing the other, constitutes an apparatus which is very easy to the patient, and very convenient to the surgeon.

But the great difficulty is in the treatment of compound fractures. No one apparatus can apply to all; the situation of the wound must in a great degree determine our choice. Fortunately, the wound is usually sufficiently in front to permit the limb to be laid on the calf. That in such a case is certainly, in our opinion, the best position. But what is the best apparatus? So far as we have seen and read, a modification of the fracture-box of Assalini supplies more than any other the requisites which we have already pointed out. The modification to which we allude is in use at this time in St. George's Hospital. It does not ascend so high at the sides as Assalini's box—above there are two pullies for fixing the ends of a bandage which passes obliquely beneath the patella, and effects the counter-extension—at the lower end is a sliding foot-board, the angle of which with the horizon also admits of alteration. The knee being fixed, the foot is tied to this by a proper tailed bandage, and foot-board and foot are forced downwards by a screw, so as to effect and keep up the extension. Between the foot and knee is a many-tailed bandage, on which the limb is laid; the tails cross in front, which is otherwise free, for the purposes of dressing and examination. Small padded splints or compresses are applied in front, if necessary, and secured to brass buttons, which are ranged along the external sides of the fracture-box. This apparatus, of which we have offered a mere imperfect sketch, we have tried and seen tried again and again. It has almost superseded all other modes of management in St. George's Hospital.

If the wound is behind, or so much on the posterior part of either side as to render access inconvenient whilst the limb is laid upon the calf, there appears to be no doubt that the best, indeed the only plan, is to place it on a splint on the side opposite to the wound.

Such are the observations we would offer on Dr. Houston's paper. We have limited ourselves to the points which he discusses, and avoided touching on many important and interesting questions. In principle we agree with Dr. Houston—that extension is the best mode of treating fractures, more particularly of the thigh. But we differ on the mode of extension he recommends in fractures of the leg.

We now turn to a method advanced with confidence, and defended with plausibility. We are struck at once with the truth of the sarcasm so frequently flung against medicine—that its certainties are such, that if one of its professors affirms a colour to be white, the next who is appealed to will probably pronounce it black. Hot and cold are blown alternately till the rational spectator is inclined to doubt the firm foundations of such a seesaw science.

We have seen Dr. Houston warmly advocating the treatment of fractures by extension. Cases have been adduced, reasoning has been employed, to set forth its merits and advantages, and not only Dr. Houston, but many other surgeons of no mean practical attainments, have assiduously laboured to invent or to improve apparatuses by which it may be best applied. But all this is an absolute delusion. Muscles, if you only let them alone, have no disposition whatever to contract; nay, so far from tending to shorten a limb, they form the nicest, softest, warmest padding for it possible. Such is the new creed of which Mr. Radley, of Newton-Abbot, in Devonshire, is the apostle. And a very ardent apostle he is. He does not mince matters—he does not say "splints may be useful in some cases," but he pronounces, without any reservation whatever, the condemnation of all splints in all cases.

Those who would desire an intellectual treat should peruse the two papers of this gentleman, contained in the Lancet for the 31st of October, and the 21st of November of last year. The papers in question are absolutely curiosities. The mixture of fancy, folly, and fun, is in keeping with this merry time of Christmas, and might be borrowed by Hood for his Comic Annual. Mr. Radley would appear to be a veteran, and his bonhommie is of the Abernethian School, though he tells us that he derived his professional lore from "the precepts of that deservedly estimated anatomist, Joshua Brookes, in his simple classification of the muscles of the extremities according to their uses, and his doctrine of muscular contraction." That those precepts and that doctrine were not instilled into the ears of the neophyte in vain we shall shortly prove. It is impossible to be out of humour with Mr. Radley, who deals his blows on the *splinters* with such good-tempered drollery, that they cannot themselves do otherwise than laugh.

It is difficult to analyse Mr. Radley's notions, as they rather border on what rhetoricians term the rigmarole. So far as we can sift them from the verbiage, they appear to form some such cinders as these.

1. Splints are unnecessary, because, says Mr. R. "do surgeons ever apply splints to a broken rib?" No, Mr. Radley, they do not; because Nature has furnished splints of her own,—the other ribs. Surgeons do, however, bandage pretty firmly for broken ribs to prevent motion of the fractured ends, (the other ribs acting like splints effect extension,) and whoever has treated many broken ribs, knows the ease which the patient usually experiences after the bandage is applied. "I have not yet heard," continues Mr. R., "that in fractures of the clavicle the inventive power of surgical genius has designed a splint." But that inventive power has designed something in this instance better than a splint—a powerful extending bandage. Suppose broken clavicles were let alone, what pretty clavicles they probably would be. Neither, says Mr. R., do we find splints employed in fractures of the lower jaw, or of the cranium. These analogies are too profound for us.

2. Splints are unnecessary, because Mr. Radley invariably succeeds without them. The only two instances of fracture of the thigh treated without splints, which Mr. Radley relates, are these.

"The next case I shall mention occurred in August, 1830, in a weak little girl, about five years old, who was being carried in the arms of her sister, when both fell together, and the younger had her thigh simply fractured. The name of the child was *Towse*, and she lived in Newton Bushel. The bones were brought into apposition on a pillow, a bandage was applied, and some isolated slips of thin mahogany *veneer*, were lightly placed here and there over the course of the fracture,—for show, not for use, not exerting any effect. My reason for placing them so was to propitiate the ignorance and prejudice which always strenuously contend for retaining old forms. I thus affected at that time to do as others did ; but not so now. The child remained under the evaporating action of a moist bandage, in an easy state, for four days, sometimes half sitting, resting on her elbow in bed, and playing with her fellows. She ate and drank as usual, and slept without disturbance, for the limb was placed in the natural position, which I in a great degree had let her choose at the first, and which she said was easy to her."

" An evil-minded old woman, on the fifth day, seeing the child so easy under a fracture of the femur, shrewdly suspected that the bone was not broken at all, and that the surgeon was only ' making a job of it.' She whispered her suspicions to another old crony (the child had lost its mother), and these wretches took off the bandage, and actually placed the child upon its feet! The bone of course was displaced. The mischief-workers saw and felt the crepitus, the child shrieked, and, conscience-stricken and alarmed, Mr. Gaye was sent for, who replaced the bandage, and, *secundum artem*, splinted it up with the pieces of veneer, though with more pressure than is consistent with my mode of procedure. The child ultimately did well; and so, indeed, do others, even where much unnecessary force is employed. Mr. Gaye, my esteemed friend, makes very good cures indeed, and uses splints; but he is extremely careful, and does not employ a tithe of that force which I have seen employed." 289.

The evil-minded old woman was wise in her generation, and so was Mr. Gaye in his. For certainly this case cannot be said to have been treated without splints, inasmuch as, after the fifth day, they were applied secundum artem by that gentleman. In the next case, no splints were used.

*Case.* " Richard Curnell, aged 45, I think, a pauper in the village of Kingsteignton, in the month of August, 1834, slid from the top of a large corn-rick which he was thatching, and fell to the ground. The height was upwards of twenty feet. The clavicle and the femoral shaft on the right side were both fractured. The fractures were not compound, but the depth of his fall and the violence of the shock greatly aggravated the symptoms. The thigh was placed on a long pillow, on a bed perfectly soft, and treated with the tailed bandage, wetted with cold lotions ; the skin was sponged with tepid water several times a day, allowed freely to evaporate in the common atmosphere of his room. As the cure advanced, camphorated and oily embrocations were applied generally over the limbs, and, at last, the supporting circular plaster of leather, overlapping the fractured portion, kept on with a bandage, perfected the cure." 289.

That a cure took place we do not doubt, but the information we should most desire would be on the length of the cured limb, and on a few other matters of that sort. Mr. Radley relates several cases of broken leg. One or two are apparently satisfactory ; the majority are of some such character as this.

*Case.* " An old Newfoundland man, residing in Kinkshenwell village, presented a somewhat parallel case. Twenty years before, and it had been my turn to serve him; he had broken his leg while engaged in the Newfoundland fishery. One night in the Christmas season of foolish carousing, he fell down on his road home, in the dark and dubious way, and again broke the same unlucky leg. This might be called act the second. His leg was treated with the same routine as my other cases, though with very humble materials. He returned to his work at the end of four weeks, and many a sailor-like oath did he bestow on 'the blockhead of a doctor' who had kept him 'so long *belayed* in board splints and blankets, in his berth, on board the old brig, on the banks of Newfoundland.'" 291.

In conclusion, Mr. Radley invokes the shade of Brookes, but, as that "can't come," he takes his old master's place, and, laying hold of a young student by the button-hole, he descants to the unhappy youth, "on some such anatomical and physiological subjects as the following."

"The radius and ulna sympathise with and support each other, in the misfortune of fracture happening to either; while, with one point of exception, from peculiarity of structure and office, the interosseous muscle and ligaments keep them in close contact, and they cannot therefore require the aid of splints. If both these bones at once are broken, be assured it will be quite natural for them, like the Siamese youths, to lie still together, unless you molest and 'bind them fast in fate,' in the painful fate of splints.

Is the humerus or the femur broken? The latter, in particular, is invested all around, and beautifully and strongly inclosed within, by integuments—by an unyielding fascia made tense at the pleasure of the will, supported by a mass of muscles, its natural defenders; with a host of vessels to supply it with warmth, and to afford the means of restoring its continuity, a continuity not lost, but merely interrupted; nerves, also, exquisitely alive to pain, which warn of the approach of danger, and will not impel their obsequious servants, the muscles, to disturb the bone, unless offended by irritating causes; and if through force or violence the bone is impelled through the investing coverings, reduce it to its place *in situ*, and all will be well, if you withhold interference with the operations of Nature—an interference that will be prejudicial, though honest.

In a fracture of the fibula we need not trouble ourselves with splinting, because its tried friend tibia will, with rest, compel it to keep its own place better than any external aid. Again, is the tibia itself broken? The interosseous ligament, when not ruptured, is a firm band of union between the two bones, just commensurate with their length. We may further quote the words of a good surgical authority:—'The fibula resists the causes that tend to produce displacement of the tibia, when fractured.' Thus much for the support given by bones in juxta-position.

But should both these bones be broken, do not fear they will be too soon placed in a condition to sustain the body. The limb in such a state is like a man who has received a stunning knock-down blow, and cannot rise again, until there is an *adequate* recovery of sensation. So the leg, if not irritated, will have a disposition to lie still until well recovered. Assist the cure, and when once settled, be careful not to inflict pain on it." 293.

The preceding may be said to be the pith of Mr. Radley's practical injunctions. He is not sanguine on the speedy prevalence of his views and treatment. "Before (he says) I shall have aroused the attention of the careless, made converts of the rich, persuaded the proud, convinced the opiniated, and compelled the interested and the obstinate to yield to the influence of shame, many years may have rolled away." Such is the inveterate obstinacy of error, that we think Mr. Radley's gloomy anticipations are very likely to be realized. For ourselves, we are not ashamed to own that we don't intend to be convinced. Our obduracy is so confirmed, that we prefer the evidence of our own imperfect senses to the eloquence of Mr. Radley—and, although we know that we ought to believe that muscles will be quiet, and not distort or shorten fractured limbs, yet such are the optical delusions we have experienced, that we cannot disburthen ourselves of the idea, that we have *seen* them do all that Mr. Radley assures us they do not. Fortunately for Mr. Radley and for truth, and unfortunately for the manufacturers of splints, the present generation will, in due season, be gathered to their fathers. Our sons will then be divested of the prejudices that now oppose our assent to the new doctrine, and some Medico-Chirurgical Reviewer, yet unborn, will pay a tribute of critical respect to the memory of the great anti-splinter.

